# Bioactive β-Carbolines in Food: A Review

**DOI:** 10.3390/nu11040814

**Published:** 2019-04-11

**Authors:** Paulina Piechowska, Renata Zawirska-Wojtasiak, Sylwia Mildner-Szkudlarz

**Affiliations:** Faculty of Food Science and Nutrition, Poznań University of Life Sciences, Wojska Polskiego 28, 60-637 Poznań, Poland; paulina.piechowska@up.poznan.pl (P.P.); mildners@up.poznan.pl (S.M.-S.)

**Keywords:** β-carbolines in food, norman, harman, neuroactivity, neurodegenerative diseases

## Abstract

Harman and norharman, two neuroactive β-carbolines, are present in several plants and in thermally processed foods. They exhibited a wide spectrum of biological and pharmacological effects, including antioxidant, neuroprotective, and anti-inflammatory effects. In this article, we review the progress of recent research on the presence of these compounds in food, as well as their various biological and neuroactive properties. Our findings strongly suggest that some foods, especially coffee, can act as a rich source of β-carbolines, which may possibly be associated with a reduced risk for serious neurodegenerative diseases, such as Parkinson’s and Alzheimer’s.

## 1. Introduction

In recent decades, many studies have focused on the bioactive constituents found in raw materials and food products. Most often, interest in these compounds is related to their antioxidative properties, which are commonly thought of as “healthy”, because oxidative processes are harmful to human health. Yet there are many natural components that have other forms of bioactivity that are sometimes even more important. These include neuroactive β-carbolines, which occur in food. This review focuses on food as a major source of β-carbolines in our diet, as well as on the neuroactive importance of these compounds.

## 2. Characteristics of β-Carbolines

β-carbolines are a group of biologically active, naturally occurring plant-derived alkaloids that are derivatives of indole. The chemical structures of the two most characteristic compounds of this group, harman (H) and norharman (NH), are related to the nonpolar heterocyclic aromatic amines, the products of pyrolysis of proteins and amino acids (they form, for example, during the heat treatment of meat) [1,2,3,4]. NH (9H-pyrido(3,4-b)indole) is considered a fundamental compound that consists of pyrrole, benzene, and pyridine rings. Harman and harmine are the most frequently studied derivatives of NH; they differ from NH in the substituents on the rings. β-carbolines are characterized by broad biological activity, possessing pharmacological and psychopharmacological properties (including antitumor, antiviral, and antimicrobial activity) [1,5,6]. The properties of β-carbolines and the ways they affect the central and peripheral nervous systems of living organisms have led to many studies attempting to determine their derivation and examining their effects [1,2,5,6]. H and NH are β-carbolines that occur endogenously in mammalian tissues and body fluids (e.g., urine and platelets) and are also stored in the organs—such as the liver and brain—of living organisms [5,7]. H is stored in large quantities in the brain and is particularly interesting. It is estimated that its level is 55 times higher in the brain than in blood plasma on account of its high lipophilicity of H, which causes it to spread much faster in the brain and the tissues of the peripheral nervous system. Carbolines are excreted in varying degrees from the body in the urine and feces, with H mostly being excreted in feces [5,6].

## 3. Occurrence of β-Carbolines

The β-carbolines norharman (NH) and harman (H) are the most frequently identified carbolines [2,8,9,10,11,12]. β-carboline compounds were first isolated from *Peganum harmala*, a species of succulents in the Nitrariaceae family. H can also be isolated from hallucinogenic plants, such as *Banisteriopsis caapi* and *Tribulus terrestris* [6]. β-carbolines have been detected in tobacco leaves, cigarettes, and cigarette smoke [9,11]. Poindexter and Carpenter recorded the level of NH and H in cigarette smoke at 12.6 μg/g and 4 μg/g, respectively. Studies of tobacco leaves have determined the presence of β-carboline at the level of 0.18–0.20 μg/g for NH and at about 0.02 μg/g for H [9]. Other levels of β-carbolines were found in the work of Tosuka et al. (1999) in six Japanese mainstream and sidestream brands of cigarettes [11]. The highest levels of NH and H were found in sidestream brands and amounted to 8990 ng/cigarette for NH and 3000 ng/cigarette for H. Herraiz (2004) obtained significantly lower values for NH and H in cigarette smoke, respectively, between 152 and 1966 ng/cigarette, and between 55 and 814 ng/cigarette [13]. In these studies, the level of NH was always higher than that of H in tobacco and cigarette smoke.

Many works have indicated the presence of NH and H in processed and stored food [1,11,13]. They can be found both in plant tissues [12,14,15] and in products of plant origin, such as bread [13], cookies [13], maize, barley, soy, coffee [2,8,16,17], coffee substitutes (such as chicory coffee) [10,18], and in fermented alcoholic beverages [13,19,20]. The presence of H and NH has also been determined in meat [11,13], fish [13], vegetables [21], fruits [21], and juices [13,21]. Due to the presence of NH and H in mammalian tissues [22], many authors have drawn attention to the possible relationship between increased levels of β-carbolines in biological fluids and human tissues and the consumption of products containing these compounds [21,23,24,25].

H and NH have been detected in Passifloraceae extracts; flowers of plants belonging to this family have been found to contain 126 ng/g d.m. H and 68.3 ng/g d.m. NH [15]. Both H and NH have been found in simple herbal medicines containing Schisandrae Fructus, Pinelliae Tuber, and Evodiae Fructus. High levels of H (0.63 μg/g) and NH (8.24 μg/g) were noted in Evodiae Fructus, which is derived from the *Tetradium daniellii* plant [15]. Both carbolines have also been detected in the dried leaves of *Tribulus terrestris* at the same level of 44 µg/g d.m. [26]. Herraiz and Galisteo (2003) determined the levels of four carboline tetrahydro-β-carboline alkaloid derivatives (1,2,3,4-tetrahydro-β-carboline-3-carboxylic acid, 1-methyl-1,2,3,4-tetrahydro-β-carboline-3-carboxylic acid, 1-methyl-1,2,3,4-tetrahydro-β-carboline, and 6-hydroxy-1-methyl-1,2,3,4-tetrahydro-β-carboline) in fruit and fruit juices. The highest concentration of 6-hydroxy-1-methyl-1,2,3,4-tetrahydro-β-carboline among fruits was found in bananas, where it amounted to 1.87 μg/g, and in tomato juice, where it was present at 2.03 mg/L. In the case of 1-methyl-1,2,3,4-tetrahydro-β-carboline, its highest level in both fruit and juice was found for kiwis at 1 μg/g and 0.3 mg/L, respectively. The highest level of 1-methyl-1,2,3,4-tetrahydro-β-carboline-3-carboxylic acid was detected in citrus fruits (1.5 μg/g in oranges and 3.5 μg/g in grapefruit) and in citrus juices (2.9 mL/L in orange juice and 2.6 mL/L in grapefruit juice). The highest concentration of 1,2,3,4-tetrahydro-β-carboline-3-carboxylic acid was found in tomato juice at 0.76 mL/L [21].

β-carbolines have also been found in cereal products. Herraiz (2004) investigated the levels of H and NH in different brands of commercial foodstuffs, including bread, toasted bread, cookies, and breakfast cereals. The level of NH ranged from below the limit of detection (0.03 ng/g) to 65.4 ng/g in bread, from 41.7 to 164.2 ng/g in toasted bread, from 7.7 to 34.0 ng/g in cookies, and from below the limit of detection (0.03 ng/g) to 187.4 ng/g in breakfast cereals. Levels of H in the tested products were lower than NH, and in toasted bread were below the limit of detection. The highest concentration of H was recorded in breakfast cereals (91.7 ng/g) [13].

β-carboline compounds have been noted in meat and fish, with their level varying depending on the type of meat and the heat treatment. NH generally occurred at higher levels. Tosuka et al. (1999) noted H and NH in different types of meat (beef, chicken, and mutton) that was heat treated by frying, flame-grilling, and oven-baking. The concentration of H in flame-grilled meat was 169 ng/g for beef, 133 ng/g for chicken, and 67.7 ng/g for mutton; in fried meat, it was only 7 ng/g for hamburgers, 3.80 ng/g for steak, 5.50 ng/g for bacon, and 0.62 ng/g for pork chops; while in oven-baked meat, the levels were 32.5 ng/g for bacon, 4.03 ng/g for roast beef, and 11.2 ng/g for pork chop. Higher values were noted for NH, whose level in flame-grilled meat was 795 ng/g for beef, 622 ng/g for chicken, and 458 ng/g for mutton; in fried meat, the value was 12 ng/g for hamburgers, 12.5 ng/g for steak, 40.2 ng/g for bacon, and 2.39 ng/g for pork chops; while in oven-baked meat, the levels were 59.6 ng/g for bacon, 5 ng/g for roast beef, and 17.3 ng/g for pork chops [11]. Herraiz (2004), studying beef and pork fillets, found that H and NH levels in raw meat were below the limit of detection. After heat treating the meat, the level of carbolines depended on the method of preparation. The highest values were recorded for well-done meat, where the range of H was between 20.7 and 32.1 ng/g and the range of NH was between 36.4 and 128.1 ng/g. Lower levels of β-carboline were found in sausages, with NH ranging from not detected to 3.7 ng/g. Carbolines were not detected in raw fish (hake, salmon, or swordfish). After treatment, the highest levels were found for cooked fish, with NH ranging from 6.4 to 48.11 ng/g and H ranging from 0.7 to 4.3 ng/g [13]. NH levels were higher than H levels, regardless of the method of preparation or type of fish [11,13].

Coffee seems to be the most important food source of β-carbolines. One of the first studies of β-carboline levels in coffee (Tsuchiya et al. 1996) [19] found the concentration to be 18.72 ng/g for H and 63.53 ng/g for NH. Herraiz (2002) investigated the concentrations of β-carbolines in prepared coffee and showed a significant variation in the levels depending on the processing stage and type of processing. Carbolines were found in green beans, roasted beans, ground coffee, blended ground coffee, decaffeinated ground coffee, instant coffee, decaffeinated instant coffee, and espresso/brewed coffee. The highest levels were found in espresso/brewed coffee, where NH ranged from 91 to 165.6 μg/g, and H varied between 26.9 and 39.9 μg/g [2]. These levels proved to be higher than in the later work of Herraiz (2004), where for espresso and brewed coffee, NH ranged between 23.6 and 165.9 ng/g and H between 5.06 and 40.86 ng/g [13]. The levels of NH and H in ground coffee and instant coffee were the same in both works. The concentrations of NH in ground coffee were 1.80 μg/g [2] and 1430 ng/g [13], while H was found at levels of 0.38 μg/g [2] and 335 ng/g [13]. Instant coffee had the highest levels of NH at 2.86 μg/g [2] and 2100 ng/g [13]. Comparison of the above data shows that, regardless of the type of coffee and the processes it underwent, NH was present at higher levels than H. The work of Alves et al. (2007) [17] determined NH and H levels in espresso coffee of several widely available brands. A comparison of the results for caffeinated blends and decaffeinated blends indicates differences in the amount of H. Levels of H were higher in caffeinated than decaffeinated blends and amounted to 2.82 μg/espresso coffee (EC) with a 30 mL infusion. This relationship could not be seen for the NH levels, which ranged between 6.9 and 7.4 μg/EC in both types of coffee. In caffeinated 100% arabica, lower levels of NH (4.10 μg/EC) were observed compared to other coffees. The study of Alves et al. (2007) [17] also determined the levels of NH and H in espresso coffee prepared with roasted arabica and robusta beans; the results indicated that the addition of roasted robusta beans (unlike the addition of roasted arabica) increased the level of NH (10.60 μg/EC), without affecting levels of H (3.64 μg/EC). In subsequent studies (Alves et al., 2010) [8], the levels of H and NH were determined for commercially available beverages made with coffee and coffee substitutes. The beverages assessed differed in the proportions of coffee, chicory, barley, malt, and rye used. The highest levels were obtained in 100% instant coffee (3.75 μg/g for NH and 1.46 μg/g for H). In the case of coffee substitutes, the highest concentrations of NH and H were found for chicory coffee (2.00 μg/g for NH and 1.34 μg/g for H). The lowest concentration of NH was determined in a barley-only coffee beverage (1.07 μg/g for NH and 0.33 μg/g for H). Coffee substitutes usually contained approximately 50% lower levels of carbolines than real coffee. The data also show that the main source of carbolines in coffee substitutes was chicory [8]. In both works of Alves et al. (2007, 2010) [8,17], similar trends to those seen for other products can be observed, in that the levels of NH are always higher than those of H. Wojtowicz et al. (2015) [10] studied traditional and new raw materials for the production of coffee substitutes and found chicory to have the highest carboline levels of traditional materials (0.69 μg/g for H and 1.25 μg/g for NH). Similarly, Alves et al. (2010) [8], examining various coffee substitutes, found the highest levels in chicory (1.34 μg/g for H and 2 μg/g for NH). Of the new substitute materials, the highest level of carbolines was found in artichoke (1.55 μg/g for H and 1.64 μg/g for NH) [10]. The addition of artichoke increased the levels of carbolines in the product. However, in another paper [18], the same authors found that roasted artichoke could also introduce high levels of other bioactive substances, including some that are undesirable for health, such as acrylamide.

At lower concentrations, β-carbolines are found in many other food products. Adachi et al. (1991) determined β-carboline levels in soy sauce, finding H to be present at 250 ng/mL and NH at 71 ng/mL [20]. Tsuchiya et al. (1996) reported much lower levels in soy sauce, at 1.56 ng/mL H and 0.29 ng/mL NH [19]. In both works [19,20], levels of H and NH were also determined for other vegetables, dairy, and starch products. Adachi et al. (1991) found the highest levels of carboline in wheat vinegar, at 730 ng/mL for H and 96 ng/mL for NH [20]. However, in the work of Tsuchiya et al. (1996), the highest concentration of carbolines among selected products was found in cocoa, at 161.8 ng/g for H and 84.42 ng/g for NH [19]. It has been shown that β-carbolines can also be found in alcoholic beverages, such as beer, wine, whiskey, brandy, sake, and vodka. Tsuchiya et al. (1996) [19] found the highest H level in sake (29.86 ng/mL), while Adachi et al. (1991) [20] noted lower values (4.1 ng/mL for H). The level of H in wine in both works was found to be similar, at 8.5 ng/mL [20] and 6.34 ng/mL [19]. The highest levels of NH were found in beer and amounted to 5.29 ng/mL [19] and 2.7 ng/mL [20].

## 4. Identification of the β-Carbolines

Norharman and harman have been found in various matrices, including food products, plant extracts, and cigarette smoke, as well as in biological fluids and tissues. Initially, thin layer chromatography (TLC) with fluorescence detection [11,26] was used to determine levels of β-carboline compounds. Adachi et al. (1991) used high-performance liquid chromatography (HPLC) with an internal standard. The products used for assays were homogenized to obtain the supernatant for analysis. An internal standard (1-ethyl-β-carboline) was added to each sample at 60 ng. Fluorescence detection was performed at an excitation wavelength of 300 nm, while for emission the wavelength was 433 nm [20]. In the work of Bourke et al. (1992) [26], a combination of the earlier TLC, UVS, and HPLC methods was used to determine NH and H in *Tribulus terrestris*. The extract was obtained from 2.5 kg of *Tribulus terrestris* and 20 L methanol. Tsuchiya et al. (1996, 1999) also used the HPLC method to determine β-carbolines in food products, cigarette smoke, and crude drugs [15,19]. The food products were homogenized and centrifuged at 10,000× *g* for 20 min. The supernatant was then filtered through 0.45 μm pores [19]. Powdered crude drug samples were also homogenized and centrifuged, though for only 15 min [15]. Tosuka et al. (1999) used the HPLC method to determine β-carboline levels. The meat was subjected to thermal treatment. To determine carbolines in meat, 5 g samples were used, homogenized with 0.1 N HCl. Fluorescence detection was performed at 260 nm for excitation and 430 nm for emission [11]. In the study of Herraiz and Galisteo (2003), to determine H and NH levels in fruit and fruit juices, reversed-phase high-performance liquid chromatography (RP-HPLC) was used. Fruits and juices of various origins, local and imported, were used. The fruits were washed and, where necessary, peeled. The 1-ethyl-1,2,3,4-tetrahydro-β-carboline-3-carboxylic acid solution was added at 5 mg/L as an internal standard to the samples (5.5 mL). Fluorescence detection was performed at 270 nm for excitation and 343 nm for emission [21].

Herraiz (2002) [2] also used the following method to analyze the β-carbolines in coffee: HPLC-MS and RP-HPLC were used to analyze selected raw materials. Coffee was prepared in a household coffee maker in two ways. First, 200 mL of water was poured over 5 g of coffee to obtain 170–190 mL of coffee. In the second method, smaller amounts of more concentrated infusion were obtained. The samples (50–60 mL) were obtained from authentic commercial espresso machines and analyzed. Instant coffee was prepared by adding 30 mL of boiling water to 2 g of instant coffee. One hundred and twenty-five microliters of a 1-ethyl-β-carboline solution (EβC) was added as an internal standard to the samples. Fluorescence detection was carried out at 300 nm for excitation and 433 nm for emission. Alves et al. (2007, 2010) [8,17] determined the level of β-carbolines in instant coffee, coffee substitutes, and espresso coffee by using the HPLC method with a harmaline solution as an internal standard. In Alves et al. (2007), β-carbolines were detected in widely available coffees obtained from local supermarkets and canteens. Espresso coffee was prepared using several selected automatic coffee machines. To 5 mL samples of diluted coffee, 50 μL of harmaline solution was added as internal standard (60 mg/L) [17]. Alves et al. (2010) prepared samples by dissolving 2 g of coffee in boiling water with the addition of 1 mL of internal standard (15 μg/mL). The β-carbolines extraction was based on the procedures described by Herraiz (2002) [8]. In the studies of Alves et al. (2007, 2010), fluorescence detection was carried out in both cases under the same conditions; the excitation and the emission wavelengths were 300 nm and 440 nm for NH and H [8,17]. The method of Alves et al. (2007) and Alves et al. (2010) was also used to determine β-carboline levels in the raw materials used to produce coffee substitute (Wojtowicz et al. 2015 and Zawirska-Wojtasiak et al. 2018) [10,18]. In those studies, a modification of the Alves method was used: sample sizes in the 1–4 g range were selected depending on the raw material. In the hawthorn fruit and artichoke samples, it was necessary to vacuum filter the supernatant because of mucilaginous substances.

## 5. Neuroactive Effects of β-Carbolines

In recent years, attention has been paid to the neuroactive effects of NH and H [27,28,29,30,31]. Like serotonin, dopamine, opioids, and benzodiazepine, H affects different receptors in the brain. Both H and NH can act as neuromodulators through their effect on monoamine oxidase (MAO), which may lead to the development and worsening of affective disorders, such as depression, Parkinson’s disease, and Alzheimer’s disease [31,32,33,34].

Parkinson’s disease involves a disturbance of the dopaminergic pathway, leading to movement disorders. Symptoms include bradykinesia, resting tremor, muscular stiffness, and postural instability. Parkinson’s disease is a progressive disease that significantly shortens the life of the patient [35]. Alzheimer’s disease is a degenerative disorder of the nervous system. It leads to a loss of neurons and synapses in the cerebral cortex and in subcortical areas. It causes general dementia, trouble with speech and memory, worsened dementia, mood swings, and ultimately leads to death. In 2015, it was estimated that Alzheimer’s disease affected nearly 47 million people around the world [36].

The enzyme MAO may play an important role in the occurrence of these symptoms. Monoaminoxidase catalyzes the transformation of biogenic amines, such as tryptamine, noradrenaline, and tyrosine, into inactive compounds, and also takes part in the metabolization of free neurotransmitters. MAO is found in most mammalian tissues, but the proportions of its different forms depend on the type of tissue: a large amount of active MAO enzyme is found in the brain. Impaired MAO function may lead to the development of various neurodegenerative diseases. Studies on β-carbolines have suggested that there may be specific MAO inhibitors. Research on the expression of this enzyme focuses on effectively inhibiting its action, which would allow treatment of both affective disorders and Parkinson’s disease [33,34,37]. Two types of MAO enzyme can be distinguished: MAO-A and MAO-B. Abnormal monoamine oxidase A function can initiate mental illness, such as depression, while changes in the monoamine oxidase B function lead to the emergence of neurological diseases, such as Parkinson’s and Alzheimer’s. The introduction of inhibitors of both enzymes would help alleviate the course of neurodegenerative and affective diseases. In healthy people, neuroprotective action can be achieved by partially inhibiting MAO by reducing the production of reactive oxygen species [33,34,37]. Studies on the activity of MAO in tobacco point to a high level of enzyme inhibitors in the form of NH and H in the brains of smokers. A correlation between elevated levels of H and NH in tobacco and cigarette smoke and the degree of inhibition of MAO has been demonstrated. The results showed a smaller percentage of Parkinson’s patients in the group of smokers [13]. Similar studies have been carried out on people who consume coffee. A negative correlation was observed between the occurrence of Parkinson’s disease and the consumption of coffee [2,38]. It was also shown that H is a potent monoamine oxidase inhibitor and stimulant, while NH has a calming effect on the body [38,39].

Studies of H and NH levels in spontaneous tremor and Parkinson’s disease have shown that an increased concentration of these β-carbolines may contribute to a milder course of neurodegenerative disease [31,32]. Oxidative stress is one of the main causes of worsening of nervous system disorders. Oxidative stress refers to an uncontrolled increase in reactive oxygen species (ROS) and the initiation of nonspecific radical additions to molecules in the cell. Oxidative stress causes structural changes and degeneration of the dopaminergic neurons responsible for dopamine synthesis. Disruption of this neurotransmitter leads to improper transmission of nerve impulses between cells. This worsens the symptoms of diseases, such as Parkinson’s and schizophrenia, by altering behavior, and also affects various functions of the brain responsible for emotions, recognition, food intake, and hormonal balance. Studies on β-carbolines have demonstrated that they have a neuroprotective effect that helps reconstruct the neurons responsible for dopamine synthesis. Attention has also been paid to the possibility of β-carbolines directly interacting with ROS, leading to them being recognized as antioxidant compounds obtainable both from natural sources and from synthesis. Their antioxidant properties can inhibit glial reactions and the formation of inflammatory states, which plays an important role in the treatment of neurodegenerative diseases [40].

β-carbolines are described as having good antioxidant, anti-inflammatory, and neuroprotective effects, while also inhibiting the initiation of apoptosis, preventing structural changes to neurons, and even contributing to the recovery of neutrons. The use of β-carbolines in neuroprotection gives hope not only of a new, effective way of treating neurodegenerative diseases, such as Parkinson’s and Alzheimer’s, but also of faster rehabilitation after stroke and brain damage. The use of H and NH can significantly improve the quality of life of people suffering from nervous system disorders [33,34,37].

Despite the many positive aspects of β-carbolines action, it is also necessary to consider the possible negative effect of these compounds. One of the most frequently discussed symptoms associated with the body’s exposure to β-carbolines is essential tremor (ET) [3,4,32]. ET is one of the most common and less well-known neurological diseases. This disorder most often affects older people, and around 9% of people over 60 are diagnosed with it; it is also called “senile tremor” [3,4]. In some cases, ET is autosomally inherited; however, in more than half of patients, it has an environmental background. Studies carried out in smokers, alcohol abusers, heroin addicts, and patients diagnosed with ET and Parkinson’s disease have shown high concentrations of H in their blood plasma. The results contribute to the study of β-carbolines, which could play an important role in the pathogenesis of these diseases [6]. The study of Louis et al. (2013) demonstrated a relationship between high β-carboline levels in the brain and the onset of ET. Their results confirmed the hypothesis: the concentration of H in the human cerebellum was 2.5 times higher than in the blood plasma, which might have contributed to the development of disorders of the peripheral nervous system [32]. Research into the structure and action of H and NH on the body showed a high molecular similarity to the neurotoxin responsible for the induction of Parkinson’s disease. β-carbolines are converted in the brain to N-methyl-β-carboline cations that have similar neurotoxicity to *N*-methyl-4-phenylpyridinium (MPP+). These cations may cause an increase in ROS and induce cell apoptosis [5]. *N*-methyl transferases in the cerebrospinal fluid have also been observed in younger patients suffering from Parkinson’s disease [5]. Metabolic biotransformations of β-carbolines help explain their presence and activity in biological fluids and tissues, as well as their participation in the pathogenesis of neurological diseases, such as ET and Parkinson’s [2,3,5].

## 6. β-Carbolines in Experiments on Laboratory Animals

Experimentation on animals allows the actions and effects of compounds to be assessed. Studies of β-carbolines have generally employed rodents (mice, hamsters, and rats) [27,29,38,41]. Carbolines were administered intraperitoneally or as an additive to fodder [29,42]. The level of carbolines was measured in the blood before and after death and in the organs [43,44,45,46]. The effects of β-carbolines on the living animal were most commonly tested behaviorally using the forced swim test, various types of the maze, and light/dark boxes [29,47,48,49].

The literature contains reports of β-carbolines having antidepressant and anxiolytic effects. Investigation of the antidepressant action of the selected carbolines in mice has shown their positive effect on animal activity in the forced swim test. After administration of harman, norharman, and harmine in doses of 5–15 mg/kg i.p. (H), 2.5–10 mg/kg i.p. (NH), and 5–15 mg/kg i.p. (harmine), it was noticed that the immobility of animals during the test was significantly reduced. These studies confirmed the antidepressant effect of the examined carbolines. This reaction was possible due to the use of an inverse agonistic mechanism in benzodiazepine receptors [41]. A significant effect of β-carbolines in modulating the animal’s memory and learning functions was noted in the work of Celikyurt et al. (2013), who examined the role played by H in the reference memory and working memory. Rats were given H at 2.5 mg/kg, 5.0 mg/kg, and 7.5 mg/kg. The results did not show that carbolines significantly affected the number of errors and delays in the reference memory. However, it the case of working memory, H in doses of 5 mg/kg and 7.5 mg/kg significantly reduced the number of errors [29]. The bioavailability of β-carbolines is an important aspect of their effect on living organisms. Li et al. (2016) conducted studies to examine metabolic profile dynamics and the pharmacokinetics of H and its metabolites in rats. H was administered to the animals, both intravenously and orally, at doses of 1 mg/kg and 30 mg/kg. The results indicated a slight intestinal absorption of H, whose total bioavailability was 19.41% via oral administration. It was suggested that the bioavailability of preparations is an important parameter to be considered when testing new drugs introduced orally and a good indicator of the drug’s entry into systemic circulation [6].

## 7. Conclusions

There is no doubt about the neuroactivity of β-carbolines, but their neuroprotective and neurodegenerative properties have not been clear to date, though more evidence seems to be available for their positive effects on health. However, negative observations are not to be neglected—on the contrary, they must be studied further and considered as part of the overall issue problem. As shown here, β-carbolines occur in various food products and are consumed in lesser or greater amount in the daily diet. The question is whether the amount consumed should be increased or not. Although cigarette smoke contains a high concentration of β-carbolines, it is hard to be recommended. The focus must thus be on food and drink—and especially on coffee and coffee substitutes, as they seem to be the richest sources. Yet coffee too is not entirely safe. No optimal solution will be available until further data is uncovered by research; however, neurodegenerative diseases are now being considered epidemiologically, and their prevention would thus seem to be the deciding point.
